# Nasal obstruction and cheek biting as overlooked sequelae of facial nerve palsy: Findings from an exploratory cohort study

**DOI:** 10.1016/j.jpra.2026.01.035

**Published:** 2026-01-29

**Authors:** Toru Miyanaga, Masanobu Yamashita, Sosuke Shima

**Affiliations:** Department of Plastic Surgery, Kanazawa Medical University, Ishikawa, Japan

**Keywords:** Facial nerve palsy, Nasal obstruction, Cheek biting, Patient-reported outcomes, Sunnybrook scale, Face scale

## Abstract

Conventional grading systems for facial nerve palsy, such as the House–Brackmann, Sunnybrook, and Yanagihara scales, assess symmetry and motor recovery but fail to capture patient-reported outcomes. Patient-reported outcome measures (PROMs), such as the Facial Clinimetric Evaluation (FaCE) scale, address this gap, yet sequelae, including nasal obstruction and cheek biting, remain undocumented.

We conducted a repeated-measures analysis within a prospective cohort study at Kanazawa Medical University. Twenty-two patients with unilateral facial nerve palsy were evaluated using paired assessments of Sunnybrook and FaCE scores obtained at multiple time points up to 1 year after onset. Objective facial function was evaluated using the Sunnybrook system, and subjective function was assessed using the FaCE questionnaire supplemented with two additional items addressing nasal obstruction and cheek biting. Symptoms were defined as scores ≤50 on a 0–100 scale.

At 1 year, nasal obstruction was reported in 9/15 cases and cheek biting in 11/15. The Sunnybrook score correlated moderately with the total FaCE score (*ρ* = 0.449, *p* = 0.003). Nasal obstruction correlated significantly with Sunnybrook (*ρ* = 0.406, *p* = 0.009) and FaCE (*ρ* = 0.597, *p* < 0.001) scores, whereas cheek biting correlated only with FaCE (*ρ* = 0.437, *p* = 0.004). Nasal obstruction was associated with all FaCE subdomains except Eye Comfort, whereas cheek biting correlated with all but Eye Comfort, Lacrimal Control, and Social Function.

This exploratory study identified nasal obstruction and cheek biting as frequent, clinically meaningful, yet overlooked, sequelae of facial palsy. Their persistence despite motor recovery underscores the limitations of physician-based grading and value of PROMs. Routine inquiry into these symptoms and their inclusion in facial palsy assessment and rehabilitation planning may enhance the comprehensiveness of evaluation and planning.

## Introduction

Facial nerve palsy is commonly evaluated using clinician-based grading systems such as the House-Brackmann, Sunnybrook, and Yanagihara scales.^1^ Among these, the Sunnybrook Facial Grading System is the most widely used and provides a reliable, objective assessment of facial function, particularly for synkinesis and motor recovery.^2^ However, these physician-based tools focus mainly on symmetry and movement, overlooking patient-reported difficulties in daily life.^3^ To address this limitation, patient-reported outcome measures (PROMs) such as the Facial Clinimetric Evaluation (FaCE) scale have been introduced to assess functional and psychosocial aspects from the patient’s perspective.^3^ Despite these efforts, specific sequelae such as nasal obstruction and cheek biting remain undocumented in current outcome systems, even though they are frequently reported in clinical practice, thereby highlighting the need for integrated frameworks that combine objective and patient-reported assessments.

Therefore, we investigated their prevalence and clinical correlations in patients with unilateral facial palsy, aiming to determine whether these underrecognized symptoms could serve as meaningful patient-reported indicators of residual dysfunction across different etiologies.

## Methods

A repeated-measures analysis, nested within an IRB-approved prospective cohort, was conducted at Kanazawa Medical University (approved by the Kanazawa Medical University Medical Research Ethics Committee, approval no I601, March 18, 2021). Written informed consent was obtained from all participants prior to study inclusion. The study complies with the Strengthening the Reporting of Observational Studies in Epidemiology (STROBE) guidelines. Twenty-two patients with facial palsy were evaluated using paired assessments of Sunnybrook and FaCE scores obtained at multiple time points up to 1 year after onset. For patients who later underwent reconstructive surgery, only preoperative data were analyzed because postoperative evaluations would reflect surgical outcomes rather than the natural recovery process. At baseline and follow-up, standardized video recordings were graded by three surgeons using the Sunnybrook system. Recordings were independently reviewed by two senior and one junior plastic surgeons, and scores were averaged. Patient-reported outcomes were assessed using the complete Facial Clinimetric Evaluation (FaCE) scale, which comprehensively evaluates six domains of facial function and quality of life. In addition, two exploratory single-item questions were added to assess nasal obstruction and cheek biting. These additional items were scored on the same 0–100 visual analog scale as the FaCE domains (0 = worst, 100 = best) to maintain internal consistency. The full wording of these added items, together with representative FaCE questions used for comparison, is shown in Supplementary File 1. As the additional items have not been formally validated, they were analyzed separately to generate hypotheses rather than to establish diagnostic criteria. A pragmatic threshold of ≤50 was provisionally defined as being indicative of symptom presence, because a score of 50 on the 0–100 scale corresponds to a response of “sometimes” or more frequently on the original five-point Likert scale. Thus, scores ≤50 reflect at least occasional occurrence of the symptom.

Spearman’s correlations were calculated between these symptoms, Sunnybrook scores, and FaCE subdomains. A p-value <0.05 was considered statistically significant. Analyses were performed using SPSS Statistics version 29.0.2.0 (IBM Corp., Armonk, NY, USA).

One-year prevalence rates were calculated. The mean scores of all FaCE subdomains were analyzed as global PRO indicators.

## Results

Twenty-two patients met the inclusion criteria: six with Bell’s palsy, eight with Ramsay Hunt syndrome, two with Bell’s or Hunt type, and six with iatrogenic palsy (parotid tumor, *n* = 4; vestibular schwannoma, *n* = 1; and mandibular fracture, *n* = 1) (Table 1). The mean age was 64.2 years, and 15 patients were male (Table 1). Correlation analyses were performed using 41 paired assessments (PRO questionnaires and Sunnybrook recordings obtained on the same day) from 22 patients. At 1-year, nasal obstruction was reported by 9/15 with available follow-up data, whereas cheek biting was reported by 11/15, respectively—rates comparable to those of the more widely recognized sequelae (Table 2).

**Table 1:** Demographic and clinical characteristics of patients with facial nerve palsy.

Case	Age	Sex	Etiology	Comorbidities
1	60	F	Bell’s palsy	HT
2	61	F	Bell’s palsy	HT
3	76	F	Ramsay Hunt syndrome	HT, DM, COPD
4	80	M	Ramsay Hunt syndrome	HT
5	72	M	Bell’s or Hunt	HT
6	27	F	Hunt	None
7	78	F	Bell’s palsy	HCV,HBV
8	77	F	Bell’s palsy	HT
9	18	M	Ramsay Hunt syndrome	None
10	50	M	Ramsay Hunt syndrome	None
11	71	M	Bell’s palsy	HT, DM
12	28	M	Ramsay Hunt syndrome	None
13	70	M	Ramsay Hunt syndrome	Liver cancer, HT
14	64	F	Bell’s or Hunt	HT
15	71	M	Ramsay Hunt syndrome	HT
16	85	M	Ramsay Hunt syndrome	HT,DM
17	66	M	Parotid tumor	Gastric ulcer
18	75	M	Parotid tumor	None
19	70	M	Parotid tumor	Pneumothorax
20	65	M	Iatrogenic	None
21	69	M	Parotid tumor	None
22	76	M	Vestibular shwannoma	HT
Mean	64.04545			

HT, hypertension; DM, diabetes mellitus; COPD, chronic obstructive pulmonary disease; HCV, hepatitis C virus; HBV, hepatitis B virus.

**Table 2:** Prevalence of symptoms at 1 year after onset in patients with facial nerve palsy.

Symptom	Rate (*N* = 15)
Eye dryness / irritation	67% (10/15)
Frequent use of eye drops/ointment	73% (11/15)
Excessive tearing	53% (8/15)
Nasal obstruction	**60% (9/15)**
Food spillage	70% (11/15)
Difficulty smiling	60% (9/15)
Cheek biting (buccal mucosa injury)	**73% (11/15)**

Correlation analysis demonstrated that the Sunnybrook score was moderately correlated with the total FaCE score (ρ = 0.449, *p* = 0.003), indicating good agreement between objective and patient-reported outcome measures. Nasal obstruction was significantly correlated with both the Sunnybrook (ρ = 0.406, *p* = 0.009) and total FaCE scores (ρ = 0.597, *p* < 0.001), whereas cheek biting showed a significant correlation only with the FaCE score (ρ = 0.437, *p* = 0.004) but not with the Sunnybrook score (*p* = 0.736) (Table 3).

**Table 3:** Spearman’s rank correlations among Sunnybrook scores, total FaCE scores, nasal obstruction, and cheek biting (*n* = 41).

	FaCE scale (Total)	
Sunnybrook	**0.449 (*p*=0.003)**	

Variable	Sunnybrook	FaCE scale (Total)
Nasal obstruction	**0.406 (*p*=0.009)**	**0.597 (*p*<0.001)**
Cheek biting	0.054 (*p* = 0.736)	**0.437 (*p*=0.004)**

Spearman’s rank correlation coefficients (ρ) are dimensionless values ranging from –1 to +1; bold values indicate *p* < 0.05.

Further analysis of FaCE subdomains revealed that nasal obstruction correlated significantly with all domains except Eye Comfort (EC), whereas cheek biting correlated with all domains except Eye Comfort (EC), Lacrimal Control (LC), and Social Function (SF) (Table 4).

**Table 4:** Spearman’s rank correlations among nasal obstruction, cheek biting, and FaCE subdomains (*n* = 41).

Variable	FM	FC	OF	EC	LC	SF
Nasal obstruction	**0.502 (*p*<0.001)**	**0.320 (*p*=0.041)**	**0.461 (*p*=0.002)**	0.164 (*p* = 0.305)	**0.515 (*p*<0.001)**	**0.498 (*p*<0.001)**
Cheek biting	**0.331 (*p*=0.034)**	**0.333 (*p*=0.033)**	**0.558 (*p*<0.001)**	0.281 (*p* = 0.075)	0.204 (*p* = 0.200)	0.209 (*p* = 0.189)

Spearman’s rank correlation coefficients (ρ) are dimensionless values ranging from –1 to +1; bold values indicate *p* < 0.05. PRO questionnaires and Sunnybrook video recordings were obtained on the same day.

Typical nasal deformities associated with Bell’s palsy are illustrated in Supplementary File 2, showing reduction of the affected nostril and narrowing of the ipsilateral nasal valve.

## Discussion

Previous studies on facial nerve palsy have consistently demonstrated a strong correlation between the Sunnybrook and FaCE scales, confirming that both reliably reflect facial motor recovery and patient-reported function.^4^, ^5^, ^6^, ^7^ This relationship was also reproduced in our analysis, supporting the validity of our dataset and consistency of our findings with those in prior literature. The present study extends this knowledge by incorporating additional PRO items targeting nasal obstruction and cheek biting—two underrecognized sequelae of facial nerve palsy. This combination of design elements represents the novel contribution of our study to the literature.

### Nasal obstruction

Nasal obstruction showed significant correlations with both Sunnybrook and total FaCE scores, confirming its validity as a symptom of facial nerve dysfunction. Among the FaCE subdomains, nasal obstruction correlated most strongly with Facial Movement (FM), Oral Function (OF), Lacrimal Control (LC), and Social Function (SF), implying that both the zygomatic and buccal branches of the facial nerve are closely involved.

Although nasal obstruction has occasionally been described in facial paralysis, the only prior quantitative studies estimated a prevalence of approximately 2%,^8^^,^^9^ whereas our PRO-based evaluation revealed a rate of approximately 70%, extending beyond flaccid cases to include spastic sequelae.

Mechanistically, nasal obstruction likely results from impaired coordination of the alar part of the nasalis and levator labii superioris alaeque nasi, together with the loss of lateral support normally provided by the buccinator. Relaxation of these muscles allows inferomedial displacement of the alar base and promotes nasal valve collapse.^8^^,^^10^ Despite their opposing mechanisms—loss of support versus over-constriction—both can lead to nasal-valve dysfunction, explaining the high prevalence observed across facial-palsy subtypes. The observed correlations between nasal obstruction, disease severity, and several FaCE subdomains further support its validity as a functional manifestation of facial-nerve impairment.

Clinically, nasal obstruction can interfere with breathing, speech resonance, and sleep quality, causing persistent discomfort in daily life. Although eye and oral symptoms are typically emphasized in facial palsy, nasal airflow and breathing function are also integral components of facial function and patient comfort.

The current FaCE scale lacks nasal-related items; hence, incorporating such domains may enhance its comprehensiveness and increase its sensitivity to real-life dysfunctions experienced by patients. A stepwise approach may be appropriate for integrating nasal symptoms into facial palsy–specific outcome assessment. The NOSE scale could first be used to confirm that nasal obstruction is attributable to facial nerve dysfunction rather than coincidental rhinologic conditions, after which nasal-related items could be incorporated into existing PROMs such as the FaCE scale.

### Cheek biting

Cheek biting correlated significantly with the total FaCE score but not with Sunnybrook, indicating a subjective and internally localized problem. As cheek biting affects the buccal mucosa—an area not visible during routine objective examination—it cannot be adequately captured by physician-based grading systems.

The strongest association was with the Oral Function (OF) subdomain, consistent with weakness or discoordination of the buccinator muscle, innervated by the buccal branch of the facial nerve. Unlike drooling or difficulty in smiling, which involve multiple branch territories (zygomatic, buccal, and marginal mandibular), cheek biting appears to be a branch-specific symptom confined to the buccal branch. The lack of correlation with Eye Comfort (EC) and Lacrimal Control (LC) supports this interpretation.

Clinically, cheek biting reflects local dysfunction of the buccinator, leading to food trapping and recurrent mucosal trauma. This symptom often causes substantial discomfort during eating and impacts quality of life.

Cheek biting is a patient-specific symptom reflecting buccinator dysfunction that cannot be assessed by physician-based grading systems. Its inclusion as an item in facial palsy–specific PROMs such as the FaCE scale may improve detection of buccal branch–specific dysfunction.

## Conclusion

Although the FaCE scale includes multiple items addressing eye and oral symptoms, it lacks questions related to nasal function. Therefore, incorporating nasal obstruction—which is anatomically and functionally linked to zygomatic and buccal branch paralysis—would be a valid enhancement for assessing facial nerve dysfunction. Likewise, cheek biting, which specifically reflects buccal branch paralysis, may serve as a valuable addition to capture branch-specific sequelae that are otherwise overlooked in existing PROMs.

Nasal obstruction and cheek biting may be underrecognized because they are internally perceived and not easily observed on routine examination, and therefore may be interpreted as arising from non-neurological factors. Our results indicate that these symptoms could contribute to an underappreciated aspect of patient-reported disability in facial palsy.

### Limitations

This study has some limitations. The two exploratory items—nasal obstruction and cheek biting—have not been validated, and their minimal clinically important differences have not been established. In addition, the small sample size limited statistical power, and the observed correlations should be interpreted as descriptive and hypothesis-generating rather than confirmatory. Further studies with larger cohorts and validated patient-reported outcome measures are warranted.

## Ethical approval

A repeated-measures analysis was conducted within an IRB-approved prospective cohort at Kanazawa Medical University (approval by the Kanazawa Medical University Medical Research Ethics Committee, approval no I601, approved on March 18, 2021). The study was conducted in accordance with the principles outlined in the Declaration of Helsinki.

## Funding

This research did not receive any specific grant from funding agencies in the public, commercial, or not-for-profit sectors.

## Declaration of competing interest

The authors declare no potential conflicts of interest with respect to the research, authorship, and/or publication of this article.

## Appendix Supplementary materials

Supplementary File 1. Full wording of the additional nasal obstruction and cheek biting items, together with representative FaCE scale questions used for comparison Image, application 1

Supplementary File 2. Typical nasal deformities observed in a patient with Bell’s palsy (A) Frontal view showing a reduction in the size of the affected (left) nostril aperture. (B) Basal view demonstrating narrowing of the ipsilateral nasal valve. The yellow dotted lines delineate the nostril aperture (A) and the vestibular opening (B) on the affected side. Image, application 2
